# Effects of Particulate Matter on the Incidence of Respiratory Diseases in the Pisan Longitudinal Study

**DOI:** 10.3390/ijerph17072540

**Published:** 2020-04-08

**Authors:** Salvatore Fasola, Sara Maio, Sandra Baldacci, Stefania La Grutta, Giuliana Ferrante, Francesco Forastiere, Massimo Stafoggia, Claudio Gariazzo, Giovanni Viegi, on behalf of the BEEP Collaborative Group

**Affiliations:** 1 Institute for Biomedical Research and Innovation, National Research Council, 90146 Palermo, Italy; stefania.lagrutta@irib.cnr.it (S.L.G.); fran.forastiere@gmail.com (F.F.); giovanni.viegi@irib.cnr.it (G.V.); 2 Institute of Clinical Physiology, National Research Council, 56126 Pisa, Italy; saramaio@ifc.cnr.it (S.M.); baldas@ifc.cnr.it (S.B.); 3 Department of Health Promotion Sciences, Maternal and Infant Care, Internal Medicine and Medical Specialities, University of Palermo, 90127 Palermo, Italy; giuliana.ferrante@unipa.it; 4 Department of Epidemiology, Lazio Region Health Service—ASL Roma 1, 00147 Rome, Italy; m.stafoggia@deplazio.it; 5 Occupational and Environmental Medicine, Epidemiology and Hygiene Department, Italian Workers’ Compensation Authority (INAIL), Monte Porzio Catone, 00144 Rome, Italy; c.gariazzo@inail.it

**Keywords:** air pollution, particulate matter, long-term exposure, random forest, questionnaire, respiratory symptoms/diseases, incidence

## Abstract

The current study aimed at assessing the effects of exposure to Particulate Matter (PM) on the incidence of respiratory diseases in a sub-sample of participants in the longitudinal analytical epidemiological study in Pisa, Italy. Three hundred and five subjects living at the same address from 1991 to 2011 were included. Individual risk factors recorded during the 1991 survey were considered, and new cases of respiratory diseases were ascertained until 2011. Average PM_10_ and PM_2.5_ exposures (µg/m^3^, year 2011) were estimated at the residential address (1-km^2^ resolution) through a random forest machine learning approach, using a combination of satellite data and land use variables. Multivariable logistic regression with Firth’s correction was applied. The median (25th–75th percentile) exposure levels were 30.1 µg/m^3^ (29.9–30.7 µg/m^3^) for PM_10_ and 19.3 µg/m^3^ (18.9–19.4 µg/m^3^) for PM_2.5_. Incidences of rhinitis and chronic phlegm were associated with increasing PM_2.5_: OR = 2.25 (95% CI: 1.07, 4.98) per unit increase (p.u.i.) and OR = 4.17 (1.12, 18.71) p.u.i., respectively. Incidence of chronic obstructive pulmonary disease was associated with PM_10_: OR = 2.96 (1.50, 7.15) p.u.i. These results provide new insights into the long-term respiratory health effects of PM air pollution.

## 1. Introduction

A recent comprehensive review of what constitutes an adverse health effect of air pollution was jointly published by the American Thoracic Society (ATS) and the European Respiratory Society (ERS) [1]. The adverse respiratory effects of air pollution span the life cycle and affect a wide range of illnesses: from symptoms like cough, sputum, wheeze, and dyspnea, to premature mortality. Morbidity, measured by hospital admissions, and prevalence, measured by the diagnoses of asthma and Chronic Obstructive Pulmonary Disease (COPD), are all related to air pollution exposure [1].

It should also be pointed out that, compared to the rest of the population, the elderly are potentially highly susceptible to the effects of outdoor air pollution due to normal and pathological aging [2].

Levels of ambient air pollution have significantly declined over the last decades in North America, Europe, and in other developed regions [3,4]. Nevertheless, epidemiological studies continue to report associations of long-term adverse effects on respiratory symptoms/diseases and lung function in adults and children, even for exposure levels below current ambient air quality standards [5,6,7,8]. Dominici et al. [6] assessed the health effects of long-term exposure to low levels of ambient air pollution, particularly below the US National Ambient Air Quality Standards (NAAQS). They observed that a 10 µg/m^3^ increase in PM_2.5_ was associated with a 7.3% increase in mortality, reaching a value of 13.6% at concentrations below 12 μg/m^3^ (PM_2.5_ NAAQS). Moreover, the health benefit per unit decrease in PM_2.5_ levels was larger for concentrations below the annual NAAQS than for those above that level [6].

There is evidence that air pollution can exacerbate existing respiratory health problems [1,9]; however, whether air pollution may also cause respiratory disease is less certain, since previous studies show conflicting results [10] with limitations on exposure assessment and on the ability to control for individual potential confounders [11]. Recently, the quality of the exposure assessment has been improved, using exposure models that combine data from air quality monitoring stations and satellite data, along with territorial data (e.g., land use) and meteorological parameters [12,13]. These new techniques allow investigating long-term health effects through individual and objective measures of air pollutants exposure.

It should be pointed out that most studies are usually based on available health data from registers or on ecological data, with a consequent approximation in the control for individual potential confounders, such as lifestyle and socioeconomic variables [11].

In this framework, the “Big data in Environmental and occupational EPidemiology” (BEEP) project, co-funded by the Italian Workers’ Compensation Authority (INAIL), was designed. The aim was to investigate the health effects of air pollution, noise and meteorological parameters on the Italian general population through integration of national data including land use, satellite, modelled meteorological fields and atmospheric composition variables, mortality records, hospitalizations, morbidity, work injuries and commuting accidents [12,14,15]. Within BEEP, a spatiotemporal machine learning model, the “random forest”, was developed to estimate daily mean PM_10_ (PM with aerodynamic diameter ≤10 microns) and PM_2.5_ (PM with aerodynamic diameter ≤2.5 microns) concentrations for each squared kilometer of Italy in the period 2006–2015 [12,16].

BEEP has given us the unique opportunity to evaluate the long-term air pollution effects on the longitudinal analytical epidemiological survey in Pisa, Italy [17,18,19,20], by linking PM levels estimated at the residential address to the individual respiratory health data, and adjusting for individual potential confounders. Indeed, this approach overcomes some limitations of the studies based on routinely collected health and environmental data.

The objective of this manuscript is to estimate the effects of particulate matter exposure on the incidence of respiratory symptoms and diseases, in a general population sample living in an area characterized by a medium-low level of air pollution. 

## 2. Materials and Methods 

### 2.1. Study Design and Population

Pisa is an urban area located few kilometers from the mouth of the Arno River, in a flat area; it is characterized by residential areas and by the presence of urban and inter-urban roads. Since 1980, the Pulmonary Environmental Epidemiology Unit of the Institute of Clinical Physiology of the Italian National Research Council (IFC-CNR) has performed epidemiological surveys to assess the effects of outdoor air pollution on human health [17,21]. A sample of subjects living in the urban/suburban area of Pisa (Tuscany, Italy) was selected using a multistage stratified family-cluster design. Detailed information on population characteristics and methods are available elsewhere [17,20,21,22,23]. Briefly, we considered the subjects participating in two surveys, performed in 1991–1993 (first survey) and in 2009–2011 (second survey). In particular, we focused on the subsample of subjects living in Pisa at the same home address in both surveys (n = 305, mean age 47.6 years at the initial interview) to minimize misclassification of the long-term exposure.

Information about symptoms/diseases and risk factors was collected through standardized interviewer-administered questionnaires developed by CNR [19,20,21,24], based on the National Heart Blood and Lung Institute questionnaire [25], for the first survey and by the EU-funded Project “Indicators for Monitoring COPD and Asthma in the EU” (IMCA II) for the second survey [26,27].

Data of fixed monitoring stations provided by the Tuscany Environmental Protection Agency showed a mean annual concentration of 25 µg/m^3^ for PM_10_ (below the EU limits: 40 µg/m^3^) in the period of the second survey. At the time of the first survey, only the total suspended particle was routinely monitored showing a mean value of about 70 µg/m^3^ [28]; considering a conversion factor of 0.7 [29], a mean value of 50 µg/m^3^ for PM_10_ mean annual concentration can thus be estimated (below the EU Council Directive 80/779: 80 µg/m^3^).

At the time of the first survey, Italian law did not require Ethical Committee approval. The protocol was approved by an Internal Review Board within the CNR Preventive Medicine Targeted Project. The second survey study protocol, patient information sheet, and consent form were approved by the Pisa University Hospital Ethics Committee (Prot. no. 23887, 16 April 2008). Further approval for the use of individual data in the statistical analyses of this manuscript was obtained by the Pisa University-Hospital Ethics Committee (Prot. no. 24567; 8 May 2018).

### 2.2. Self-Reported Risk Factors

Self-reported risk factors were assessed both in the first and in the second survey. Risk factors were defined as follows:

- Age: difference, computed in years, between the date of issue of the questionnaire and the date of birth (both surveys);
- Smoking status, defined according to the following categories:
  —non-smokers: subjects who had never smoked any kind of tobacco (first survey), or those who had never smoked for at least one year (second survey);
  —smokers: subjects who currently smoked (first survey), or those who currently smoked at least one cigarette daily (second survey);
  —ex-smokers: those who had smoked before the examination, but did not smoke at the moment of the examination (both surveys);
- Occupational exposure: exposure to fumes, gases, dusts or chemicals in the working environment during lifetime (both surveys).

### 2.3. Particulate Matter (PM) Exposure Levels

For each individual residential address, annual mean concentrations of PM_10_ and PM_2.5_ for the year 2011 were estimated through a Random Forest Machine Learning Approach (RFMLA); the entire process is fully described elsewhere [12,15,16]. Briefly, for each day of the years between 2006 and 2015, and for each squared kilometer of Italy, we collected several spatial and spatiotemporal parameters. Spatial parameters included: geo-climatic zone, resident population, point emission sources, total emissions, mean elevation, imperviousness surface areas, light at night data, land cover data, road density data, proximity to airports, ports, sea, lakes. Spatiotemporal parameters included: PM monitored data from all the available monitoring sites, Aerosol Optical Depth (AOD) data from the NASA Multi-Angle Implementation of Atmospheric Correction (MAIAC) algorithm, daily mean air temperature, sea-level barometric pressure, precipitations, relative humidity, wind speed and direction, planetary boundary layer height, Normalized Difference Vegetation Index (NDVI), and desert dust advection days. 

We therefore trained a four-stage model to predict daily PM_10_ (2006–2015) and PM_2.5_ (2011–2015) concentrations for each 1 × 1 km (according to the International System of Units, SI) grid cell:

- Stage 1: expansion of the database of PM_2.5_ monitors by borrowing information from the co-located PM_10_ data;
- Stage 2: imputation of missing MAIAC-AOD data using co-located multi-band Copernicus Atmosphere Monitoring Service (CAMS) data;
- Stage 3: calibration of the spatiotemporal PM concentrations with the spatial and spatiotemporal parameters;
- Stage 4: prediction of PM over all 1 km^2^ grid cells of Italy using the output of the Stage 3 model.

The accuracy of the measurements was evaluated through a 10-fold cross-validation approach, by estimating the R^2^, i.e., the percentage of variability of the observed PM values captured by the predictions. Cross-validated R^2^ were 0.71 and 0.75 for PM_10_ and PM_2.5_, respectively, demonstrating fair predictive properties [12].

The daily series of exposure levels estimated on the grid cells were linked to the residential addresses of the subjects according to their spatial locations, and the annual average exposure levels were calculated for the year 2011 (since this was the first year with available estimates for both PM_10_ and PM_2.5_).

### 2.4. Respiratory Symptoms/Diseases

Respiratory symptoms/diseases were defined as follows:

- Asthma, if the subjects reported asthma confirmed by a physician (both surveys);
- Rhinitis, if the subjects reported hay fever or other conditions causing runny or blocked nose, apart from common colds (first survey) or if the subjects reported hay fever or problems with sneezing or a runny or blocked nose, apart from common colds (second survey);
- Chronic phlegm, if the subjects reported phlegm apart from common colds at least three months of the year for at least two years (both surveys);
- COPD diagnosis, if the subjects reported chronic bronchitis or emphysema confirmed by a physician (first survey) or if the subjects reported chronic bronchitis, emphysema or COPD confirmed by a physician (second survey).

For each symptom or diagnosis, the cumulative incidence was computed as the proportion of subjects who reported the outcome of interest in the second survey (“incident cases”) over the number of subjects who had not reported the outcome of interest in the first survey (“population at risk”).

### 2.5. Statistical Analysis

The characteristics of the study sample at the two surveys were summarized as means and standard deviations (SDs) for quantitative variables and numbers (No.) and percentages (%) of subjects for categorical variables. Comparisons between the two surveys were performed through paired *t*-test for quantitative variables and Stuart–Maxwell test for categorical variables (a paired version of the more common Chi-squared test) [30]. Median, 25th and 75th percentiles were reported for the estimated PM levels.

The joint effect of self-reported risk factors and PM levels on the incidence of each respiratory disease was estimated through multivariable logistic regression models. Due to the low number of new events, the Firth’s method was used. This approach is indeed recommended in case of sparse data to ensure finite and unbiased point estimates; in this framework, confidence intervals can be derived by considering the penalized likelihood ratio as the pivotal statistic [31]. Moreover, backward stepwise variable selection was performed to obtain parsimonious models and improve the statistical power. The risk factors ascertained during the first survey (1991–1993) were included in the models; a sensitivity analysis was performed by considering the risk factors ascertained at the second survey (2009–2011). The results were expressed as odds ratio (OR) and 95% confidence interval (CI). The significance level was set at 0.05. All the statistical analyses were performed through R version 3.6.1 (R Foundation for Statistical Computing, Vienna, Austria); the R package brglm [32] was used to apply the Firth’s method.

## 3. Results

**Figure 1:**
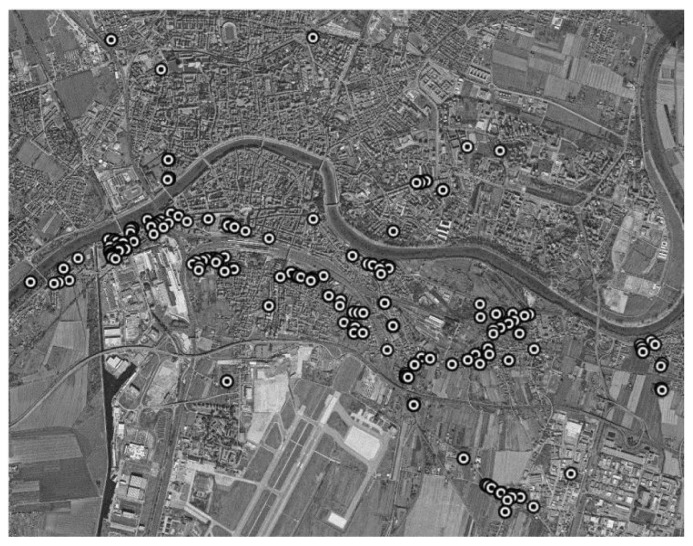
Residential locations of the study sample.

Figure 1 depicts the spatial distribution of the subjects’ residences in the study sample.

Subject characteristics at the two surveys are summarized in Table 1. The mean age was 47.6 years at the first survey and 64.2 at the second survey. A total of 135 subjects (44.3%) were males and 170 (55.7%) were females. Seventy-six subjects (24.9%) reported to be current smokers at the first survey; the distribution of the smoking status changed significantly at the second survey (*p* < 0.001), when the number of current smokers decreased to 45 (14.8%). A total of 135 subjects (44.3%) reported an occupational exposure during life at the first survey, while 125 (41.0%) reported this risk factor at the second survey (*p* = 0.307). The median (25th–75th percentile) exposure levels for the year 2011 were 30.1 µg/m^3^ (29.9–30.7 µg/m^3^) for PM_10_ and 19.3 µg/m^3^ (18.9–19.4 µg/m^3^) for PM_2.5_.

Table 2 reports the results of the logistic regression models when considering the risk factors ascertained at the first survey. Incidence of rhinitis (90/264 = 34.1% overall) was significantly associated with increasing PM_2.5_ (OR = 2.25 (95% CI: 1.07, 4.98) per unit increase (p.u.i.)). Incidence of COPD (29/282 = 10.3% overall) was significantly associated with PM_10_ (OR = 2.96 (1.50, 7.15) p.u.i.), as well as with age (OR = 1.87 (1.29, 3.02) per 10-year increase), and current smoking (OR = 2.99 (1.08, 9.39) vs. never smoking); moreover, it was not significantly associated with occupational exposure (OR = 1.91 (0.83, 4.79) vs. not exposed). Incidence of chronic phlegm (16/262 = 6.1% overall) was linked to increasing PM_2.5_ (OR = 4.17 (1.12, 18.71) p.u.i.) and occupational exposure (OR = 5.41 (1.88, 21.79) vs. not exposed). Even if reported for completeness, overall asthma incidence was too low (4/284 = 1.4% overall) to yield reliable estimates.

A sensitivity analysis was performed considering the risk factors ascertained at the second survey. The results remained substantially unchanged, except for the exclusion of smoking status and occupational exposure from the COPD model and the inclusion, in the same model, of male gender, even if the relevant effect was not statistically significant (OR = 2.16 (0.98–5.04)). Moreover, although PM_2.5_ was included in the chronic phlegm model, its effect was no more statistically significant.

**Figure 2:**
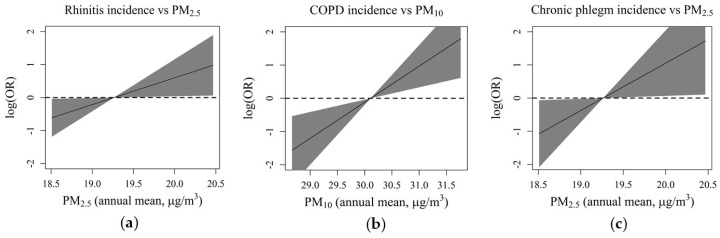
Exposure-response functions: (**a**) PM_2.5_ vs. rhinitis incidence; (**b**) PM_10_ vs. COPD incidence; (**c**) PM_2.5_ vs. of chronic phlegm incidence. The log-ORs were calculated assuming the median annual concentrations of PM as the reference (30.1 µg/m^3^ for PM_10_ and 19.3 µg/m^3^ for PM_2.5_).

Figure 2 represents the estimated exposure-response functions for PM and the incidence of respiratory symptoms/diseases; the log-ORs were calculated assuming the median annual concentrations of PM as the reference (30.1 µg/m^3^ for PM_10_ and 19.3 µg/m^3^ for PM_2.5_).

## 4. Discussion

Our study has provided new insights into the incidence of respiratory symptoms and diseases related to long-term exposure to air pollution in the city of Pisa, Italy. Indeed, exposure to PM was associated with new onset of rhinitis, COPD and chronic phlegm, after taking into account the main individual risk factors (age, sex, smoking habits and occupational exposure). These results pertain to a general population sample living in an urban area characterized by mean annual levels of PM below the current ambient air quality standards of the European Union.

Since 1990, the prevalence of rhinitis has been increasing worldwide [33]. This increase was previously shown in the Pisa sample with prevalence values ranging from 16% in 1985–1988 to 37% in 2009–2011 [19] and with a cumulative incidence value of 32% from 1991–1993 to 2009–2011 [20]. Possible determinants of this upward trend are: increasing air pollution, poor indoor air quality, improved hygiene practices and climatic changes [34,35,36,37].

Indeed, in our study, an increase of 1 µg/m^3^ in PM_2.5_ annual mean was associated with a higher risk of developing rhinitis (OR 2.25). This result is in line with our previous data showing a strong relationship between incident rhinitis and incident exposure to a proxy of outdoor pollution, i.e., vehicular traffic near home (OR 1.8) [20].

Relatively few studies evaluated the link between air pollution and rhinitis [38,39,40]. The first study about the effect of long-term PM exposure and rhinitis incidence in adults was published in 2018: data from two European cohort studies did not show any association between annual exposure to NO_2_, PM_10_ and PM_2.5_ at the participants’ home addresses and rhinitis incidence reported by the subjects [39]. Differently, the same authors later on observed that, among participants with no allergic sensitization, increase in NO_2_, PM metrics and traffic exposure were linked to an increased severity score of rhinitis with an exposure-response relationship; differently, among participants with allergic sensitization, increase in air pollution exposure was associated with an increased severity score of rhinitis only for PM_2.5_ [40].

With regard to asthma, our study had very few cases of incident asthma due to the relatively old mean age of our population, which did not let us make any inference for a possible link with air pollution. It is to point out that a recent review [41] identified ten studies in adults that assessed the association between long-term exposure to air pollution and incident asthma, and concluded that adult never/former smokers seem to be at higher risk of incident asthma due to air pollution. 

As far as bronchitis symptoms/diseases are concerned, we observed that an increase of 1 µg/m^3^ in PM_2.5_ annual mean value was associated with a higher risk of chronic phlegm incidence during the 18-year follow-up (OR 4.17).

In the literature, there are inconsistent results on the possible relationship between classically defined chronic bronchitis (chronic productive cough) and long-term exposure to air pollutants. A recent study on 50,000 U.S. women [42] showed associations of chronic bronchitis prevalence with PM_10_ and PM_2.5_ exposure; however, there was no association with chronic bronchitis incidence, possibly due to the relatively short follow-up duration (mean: six years). From another point of view, a Swiss study showed that declining levels of PM_10_ were associated with fewer new reports of regular phlegm (OR 0.74) during a 10-year follow-up [43], suggesting a relationship between changes in air pollution and bronchitic symptom development or reduction.

Concerning COPD, in our population, an increase of 1 µg/m^3^ in PM_10_ annual mean was associated with a higher risk of such disease (OR 2.96).

Indeed, recent studies reported that outdoor air pollution has long-term effects on lung function [44,45]. In particular, higher pollution exposure leads to accelerated lung function decline in general population cohorts, possibly contributing to the development and progression of the disease [44]. Positive associations between COPD incidence and air pollutants exposure were recently found in other studies. Data derived from a UK cohort showed a significant association between increasing PM_2.5_ (every 1.9 μg/m^3^) and PM_10_ (every 3 μg/m^3^) at home address level (1 × 1 km spatial resolution) and the risk of developing COPD, as defined by physician diagnosis, with hazard ratios (HR) of 1.12 and 1.10, respectively, over a four-year follow-up [46]. A more recent study on Australian women observed an association between the log of PM_10_ exposure within 10 km of residence and COPD reported incidence (HR 1.024) over a 20-year follow-up [10]. In a Taiwanese cohort, the association between two-year average ground concentration of PM_2.5_ at home address (1 × 1 km spatial resolution) and physician-diagnosed COPD development was analyzed after a mean follow-up of six years on an adult cohort: subjects exposed to the fourth (>31.86 μg/m^3^), third (23.94–31.86 μg/m^3^), and second (21.42–23.94 μg/m^3^) quartiles of PM_2.5_ had a HR of 1.23, 1.30 and 1.39 for COPD development, respectively [47].

Some study limitations should be acknowledged. The use of questionnaires for collecting information on symptoms/diseases might be a limitation because it is potentially affected by a reporting bias, as it relies upon individual memory. Nevertheless, the standardized questionnaire is one of the main investigation tools in respiratory epidemiology [48,49]. It is to point out that the questionnaire used in the second survey was slightly different, but only questions that were comparable or identical to the questionnaire used in the first survey were chosen. Moreover, a sensitivity analysis was performed, by considering the individual risk factors reported at both the first and the second survey. The results confirmed those found in the main analyses, except for the association between PM_2.5_ and chronic phlegm, no more reaching the statistical significance.

**Figure 3:**
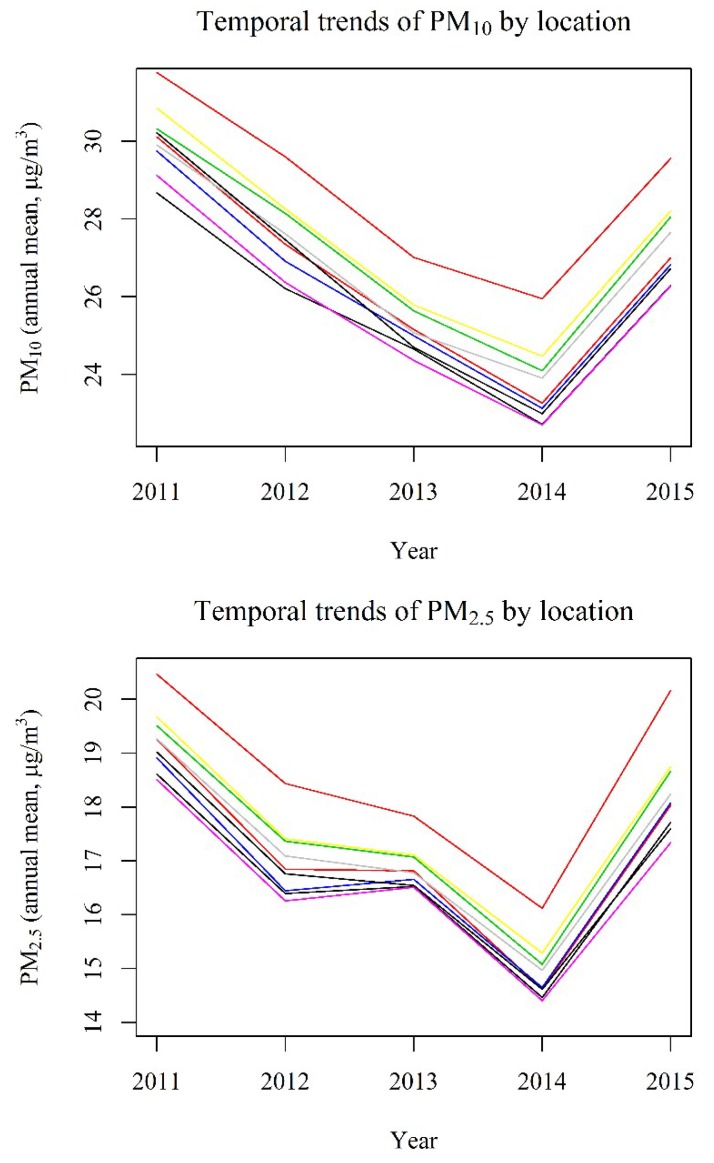
Observed temporal trends for the annual mean concentrations of PM from 2011 to 2015, in 10 randomly selected residential locations.

The annual average exposure levels were calculated for the year 2011, i.e., only at the time of the second survey, since this is the first year with available estimates for both PM_10_ and PM_2.5_. For the latter, in fact, the monitoring systems were installed quite recently in Italy. Despite the average PM_10_ concentrations decreased over the period 1991–2011 (from about 50 μg/m^3^ to 25 μg/m^3^), we hypothesized that the relative differences (spatial contrasts) in the average PM exposure levels among the locations (residential addresses) remained approximately constant. Indeed, we represented the observed temporal trends for the annual mean concentrations of PM from 2011 to 2015, in 10 randomly selected residential locations (Figure 3), and observed that the vertical differences among the curves remained approximately constant over the years. The aforementioned hypothesis is reinforced by several previous studies. As reported by Hoek, the spatial stability of air pollution contrasts is a necessary assumption for application of recently developed models for long-term exposure [50]. The same author observed that, for traffic-related pollutants including PM, spatial stability can be expected [50]. Moreover, the assumption that within-city spatial patterns remain constant over the years, also when mean concentrations of air pollutants change over time, has been made within the European Study of Cohorts for Air Pollution Effects (ESCAPE) [51]. Similar between-city spatial patterns were also observed in the Study on Air Pollution and Respiratory Diseases in Adults (SAPALDIA) [52] and in the Harvard Six Cities [53] cohorts. Even in the European Community Respiratory Health Survey (ECRHS), the authors used air pollution estimates only at the time of the second survey in order to assess relationship with the incidence of respiratory outcomes [54].

Another limitation is the exposure assessment on the home address only, without taking into account exposures related to other exposure paths (daily commuting, other indoor microenvironments). Future studies might benefit from the integration of various sources of exposures to provide a thorough overview of the effects of air pollution on respiratory health.

Among the study strengths, we can highlight the use of an advanced statistical methodology, based on a RFMLA integrating satellite data and land use variables, to estimate the annual mean concentrations of PM_10_ and PM_2.5_ for each residential address. Indeed, random forests are one of the most powerful tools to obtain predictions for continuous or categorical variables, as they correct for the overfitting tendency of decision trees in the training set.

Another strength is to have applied, over an 18-year follow-up, the same study design, sampling frame and study protocol in repeated cross-sectional surveys on general population samples living in the same area.

This study, integrating individual data from questionnaires with individual measures of PM exposure, has allowed assessing the relationship between air pollutants exposure and respiratory health, after controlling for individual potential confounders like age, sex, smoking habits and occupational exposure. However, due to the small sample size with relatively few symptom/disease onsets, there was the need to apply a stepwise variable selection procedure in order to obtain a parsimonious model and increase the statistical power; as a consequence, some independent variables were excluded from the analyses. We plan to overcome this limitation in a new multi-center study that is about to start.

## 5. Conclusions

This study adds new evidence about the effects of long-term exposure to PM_2.5_ and PM_10_ on the incidence of rhinitis, chronic phlegm and COPD in adults living in an urban area. Such negative health effects of particulate air pollution emerged in a general population sample living in an area with a concentration of air pollutants well below the current legal standard. 

This study provided estimates of the health effects in an urban population of long-term residents, using PM levels estimated at the residential address and adjusting for individual potential confounders. This let us overcome some limitations of the studies based only on registers or routinely collected health (e.g., mortality and hospitalization) and environmental data.

The different associations of PM_10_ and PM_2.5_ with different respiratory disorders, found in the current study, may be important for suggesting further research directions, which may also be helpful to policy makers when issuing regulatory decisions.

## Figures and Tables

**Table 1:** Characteristics of the study sample at the two surveys.

Characteristics	First Survey (1991–1993) n = 305	Second Survey (2009–2011) n = 305	*p*-Value ^1^
Age, years, mean (SD)	47.6 (15.6)	64.2 (15.6)	**<0.001**
Gender, No. (%)			1.000
Male	135 (44.3)	135 (44.3)	
Female	170 (55.7)	170 (55.7)	
Smoking status, No. (%)			**<0.001**
Non-smoker	142 (46.6)	144 (47.2)	
Ex-smoker	87 (28.5)	116 (38.0)	
Current smoker	76 (24.9)	45 (14.8)	
Occupational exposure, No. (%)	135 (44.3)	125 (41.0)	0.307
PM_10_, µg/m^3^, median (25th–75th percentile) ^2^	-	30.1 (29.9–30.7)	-
PM_2.5_, µg/m^3^, median (25th–75th percentile) ^2^	-	19.3 (18.9–19.4)	-

^1^*p*-value is from paired *t*-test for quantitative variables and Stuart–Maxwell test for categorical variables. Significant *p*-values are reported in bold. ^2^ Estimated exposure levels at the residential address for the year 2011, 1 km^2^ resolution.

**Table 2:** Associations (odds ratio, OR, and 95% confidence intervals (CI)) between risk factors ascertained during the first survey (1991–1993) and cumulative incidences of asthma, rhinitis, Chronic Obstructive Pulmonary Disease (COPD) and chronic phlegm ascertained at the second survey (2009–2011), from multivariable logistic regression models with Firth’s correction.

	Asthma	Rhinitis	COPD	Chronic Phlegm
Cumulative incidence:	4/284 (1.4%)	90/264 (34.1%)	29/282 (10.3%)	16/262 (6.1%)
Independent variables:	OR (95% CI)	OR (95% CI)	OR (95% CI)	OR (95% CI)
PM_10_ (1 µg/m^3^ increase) ^1^	- ^2^	- ^2^	**2.96 (1.50–7.15)**	**- ^2^**
PM_2.5_ (1 µg/m^3^ increase) ^1^	- ^2^	**2.25 (1.07–4.98)**	- ^2^	**4.17 (1.12–18.71)**
Age, years (10-year increase)	- ^2^	- ^2^	**1.87 (1.29–3.02)**	- ^2^
Male gender	- ^2^	- ^2^	- ^2^	- ^2^
Smoker (ref = non-smoker)	**12.96 (1.25–∞)**	- ^2^	**2.99 (1.08–9.39)**	- ^2^
Ex-smoker (ref = non-smoker)	4.86 (0.27–∞)	- ^2^	1.67 (0.60–4.89)	- ^2^
Occupational exposure	- ^2^	- ^2^	1.91 (0.83–4.79)	**5.41 (1.88–21.79)**

^1^ Estimated exposure levels at the residential address for the year 2011, 1 km^2^ resolution. ^2^ Variables excluded by the stepwise selection procedure. Significant odds ratios are reported in bold.
